# Gender differences and determinants of health related quality of life in coronary patients: a follow-up study

**DOI:** 10.1186/1471-2261-11-24

**Published:** 2011-05-27

**Authors:** María Dueñas, Carmen Ramirez, Roque Arana, Inmaculada Failde

**Affiliations:** 1 Área de Medicina Preventiva y Salud Pública. Universidad de Cádiz. Spain; 2 Servicio de Cardiologia, Hospital Universitario "Puerta del Mar". Spain

**Keywords:** Gender, HRQL, SF-36, Coronary patients

## Abstract

**Background:**

The role of gender differences in Health Related Quality Life (HRQL) in coronary patients is controversial, so understanding the specific determinants of HRQL in men and women might be of clinical importance. The aim of this study was to know the gender differences in the evolution of HRQL at 3 and 6 months after a coronary event, and to identify the key clinical, demographic and psychological characteristics of each gender associated with these changes.

**Methods:**

A follow-up study was carried out, and 175 patients (112 men and 63 women) with acute myocardial infarction (AMI) or unstable angina were studied. The SF-36v1 health questionnaire was used to assess HRQL, and the GHQ-28 (General Health Questionnaire) to measure mental health during follow-up. To study the variables related to changes in HRQL, generalized estimating equation (GEE) models were performed.

**Results:**

Follow-up data were available for 55 men and 25 women at 3 months, and for 35 men and 12 women at 6 months. Observations included: a) Revascularization was performed later in women. b) The frequency of rehospitalization between months 3 and 6 of follow-up was higher in women c) Women had lower baseline scores in the SF-36. d) Men had progressed favourably in most of the physical dimensions of the SF-36 at 6 months, while at the same time women's scores had only improved for Physical Component Summary, Role Physical and Social Functioning; e) the variables determining the decrease in HRQL in men were: worse mental health and angina frequency; and in women: worse mental health, history of the disease, revascularization, and angina frequency.

**Conclusions:**

There are differences in the evolution of HRQL, between men and women after a coronary attack. Mental health is the determinant most frequently associated with HRQL in both genders. However, other clinical determinants of HRQL differed with gender, emphasizing the importance of individualizing the intervention and the content of rehabilitation programs. Likewise, the recognition and treatment of mental disorders in these patients could be crucial.

## Background

Coronary heart disease (CHD) is the leading cause of death and disability among both women and men [1,2]. However, coronary disease affects men and women differently. Men suffer four times as many coronary events as women,[3] but the first episode of an acute myocardial infarction is more likely to be fatal in women. In recent years, epidemiological studies have demonstrated that women with coronary disease often remain undiagnosed, or if diagnosed, the severity of the illness is frequently underestimated [4]. Furthermore, compared to men, women return to work and use cardiac rehabilitation programmes less frequently [5].

It has been reported that women with coronary disease are older, have a higher burden of comorbid illnesses,[6] are more often widowed or living alone, have more depressive symptoms, and have poorer psychosocial adjustment following a coronary event [7-10]. However, the reasons why the disease affects men and women differently remain unclear.

Health-related quality of life (HRQL) is a commonly used measure of results for defining health in terms of both how individuals feel (distress and well-being) and how they evaluate their health and prospects for the future. Different studies performed in the general population have shown that HRQL is worse in women than in men [1,11-19]. Likewise, several authors have shown that women with coronary disease report significantly poorer physical functioning and mental health than men [5,11,16,20,21].

Different factors have been associated with HRQL in patients with coronary heart disease. Brink et al [1] reported that depression measured at 1 week after an acute myocardial infarction predicted women's physical health (PCS) at 1 year. Other authors have shown that smoking, regular alcohol consumption, and overweight are the most common risk factors for worse HRQL in men, while psychological distress, role pressure, and less strenuous exercise are more characteristic of women [22].

Prior data suggest that women with cardiac disease are more likely than men to be confronted with continuing demands in the home environment, and may be more likely to neglect health care needs [17]. Thus, Emery et al [17] hypothesized that quality of life would be more strongly associated with social support among women than among men.

Despite all the considerations above, it is necessary to improve the knowledge of the gender differences in HRQL and the effect that sociodemographic, clinical and psychological variables have on the evolution of HRQL after a coronary attack in men and women. Therefore, we carried out this study to know the gender differences in the evolution of HRQL at 3 and 6 months after a coronary event, and to identify the key clinical, demographic and psychological characteristics associated with these changes for each gender. Thus, it was hypothesized that the factors determining the evolution of HRQL in men and women would be different. It was further hypothesized that HRQL would be strongly associated with mental health, especially in women.

## Methods

A follow-up study was carried out in the cardiology unit of a university hospital in the south of Spain (800 beds), where 186 consecutive patients admitted for a suspected acute episode of coronary heart disease were identified. 175 of the patients were diagnosed with acute myocardial infarction (AMI) or unstable angina on the basis of clinical, biochemical and electrocardiographic criteria, and on the basis of their hospital discharge report. Patients with non-ischemic or non-cardiological precordial pain were excluded.

Patients were considered to have had an AMI if they met at least 2 of the following criteria: precordial pain lasting 20 minutes or more; CK (creatine kinase) and CK-M (creatine kinase-muscle) above normal values in at least 2 serum samples; and/or the appearance of the Q wave in at least 2 ECG (electrocardiography) readings.

Patients were classified as having unstable angina if they suffered precordial pain similar to the first group, and showed changes in the ST segment of the ECG, without a high enzyme level.

A previously-trained interviewer, who was not the cardiologist who made the clinical evaluation, obtained the information at baseline (9 days; SD: 7.5) after admission when the patient was clinically stable, and 3 months and 6 months after discharge. Before inclusion, all the patients were asked for their informed consent and agreed to participate (n = 175).

The study was conducted in agreement with the Helsinki Declaration and with standard working procedures and protocols, and it was approved by the Clinical Research Ethics Committee at the hospital, ensuring adherence to the norms of good clinical practice.

Sociodemographic and clinical information was obtained at baseline from a structured questionnaire and from the patients' clinical records. These variables were cardiovascular risk factors (consumption of tobacco, hypertension, hypercholesterolemia, obesity, physical activity and diabetes) and clinical information (previous history of coronary heart disease, ejection fraction, diagnostic group). The existence of comorbidity was assessed if another chronic pathology (digestive, respiratory, osteomuscular, neurological or of another nature) was explicitly documented in the patients' clinical records.

HRQL was assessed at baseline, 3 and 6 months using the eight specific and the two Physical and Mental Component Summaries (PCS and MCS) of the SF-36v1 health questionnaire. Each of the eight dimensions of the questionnaire (PF: physical functioning; RP: role physical; BP: body pain; GH: general health; VT: vitality; SF: social functioning; RE: role emotional; MH: mental health) were coded, aggregated and transformed into a scale from 0 (the worst state of health for that dimension) to 100 (the best state of health). The summary indices (PCS and MCS) were calculated using a z-score transformation for each dimension using the means and standard deviations in the SF-36 of the Spanish population. Then, the aggregate scores for the physical and mental component scale scores were computed. In the case of the PCS this involved multiplying each SF-36 scale z-score by its respective factor score coefficients. The MCS scores were calculated in the same way. Finally, these scores were standarized to a T-score where the mean was set to 50 and the SD to 10 [23].

Mental health was also measured 3 times (baseline, 3 and 6 months) during follow-up using the GHQ-28 (General Health Questionnaire), an instrument developed as a screening method to detect non-psychotic psychiatric disorders. The 28-item version was translated into Spanish and validated by Lobo et al [24] and has already been validated as a means of detecting problems in cardiology patients [25]. The questionnaire consists of 28 items and the score in the scale ranges between 0 and 28 points, a higher score indicating a higher probability of mental disorders. The cut-off point recommended for the questionnaire is ≥6 points, thus providing a sensitivity of 76.9% and a specificity of 90.2% [24].

The variables rehospitalization, return to work and frequency of angina were all assessed twice during follow-up (3 and 6 months). However, to consider these variables in a longitudinal form a variable was created taking the value 0 in all patients as baseline data. With the information from this variable and the 2 assessments carried out (3 and 6 months), a new variable was constructed and it was coded as 0: no event occurred to the patients during the whole period, 1: the event occurred to patients during the first 3 months, and 2: the event occurred to the patients between 3 and 6 months.

The revascularization treatment was evaluated three times during follow-up. As with the variables mentioned above (rehospitalization, return to work and frequency of angina), a new variable was constructed with the information from the 3 assessments (baseline, 3 and 6 months) of the revascularization treatment and this new variable was coded as 0: patients not undergoing revascularization during the whole period, 1: patients undergoing revascularization during admission, 2: patients undergoing revascularization during the first 3 months after discharge, and 3: patients undergoing revascularization between 3 and 6 months after discharge.

### Statistical Analysis

A descriptive analysis was conducted, and the chi-square test and Student t-test were performed to compare the characteristics of the men and women responding and not responding at 3 and 6 months of follow-up.

Likewise, as a previous exploratory analysis, a repeated measures ANOVA and Friedman's test were used to assess the changes in SF-36 scores in each gender during follow-up, using the Bonferroni test and Wilcoxon test for *post hoc *comparisons. A result was considered statistically significant if the p value was < 0.05.

To assess the evolution of each dimension of the SF-36 questionnaire during the study, and to find out which sociodemographic and clinical variables affected this evolution, a regression model using Generalized Estimating Equations (GEE) was constructed for each gender and for each dimension. These models are an extension of generalized linear models, constructed to produce more efficient estimates than ordinary least squares regression in repeated measures studies because they account for correlations between observations. GEE models are more flexible than other models as they allow subjects with incomplete data to be included in the analyses. If a particular subject is missing one or more out of T repeated measurements, the remaining available data from the other measurements for that particular subject are used in the analyses [26]. Also, the dependent variable in the model can have a distribution different to the normal distribution and the predictor variables included in the models can be continuous variables, ordinals or categorical variables [27]. These models have not been extensively used in healthcare research.

The dataset is presented in longitudinal form, where for each patient there were as many registers as evaluations. The dependent variable included in each model was the corresponding dimension's score at the 3 times studied for each gender, and the independent variables were: the follow-up time (baseline, 3 and 6 months), sex, age, educational level, diagnostic group (AMI versus unstable angina), previous history of coronary heart disease, ejection fraction, comorbidity and risk factors. The variables rehospitalization, return to work, revascularization and angina frequency were introduced into the models in their new, recoded form, as stated above, and the GHQ-28 score was entered as a time-varying covariate. The criteria used for selecting the co-variables included in the models were statistical (significance observed in the bivariate analysis, *P *< .05) and the clinical criteria recognized in the literature. To select the best model, the parameters of goodness of fit were used. The parameters were expressed by "quasi-likelihood under the independence model criterion" (QIC) and "corrected quasi-likelihood under the independence model criterion" (QICC), with the lowest possible values [28]. The analysis was performed using the SPSS.v17 program.

## Results

Of the 175 patients who were initially included in the study, 112 (64%) were men and 63 (36%) were women. Around 90% of both the men and women had one or more cardiovascular risk factors, arterial hypertension (52.7% of men and 68.3% of women) being the most common. Likewise, the presence of comorbidity or a previous history of coronary disease was observed in over 50% of the patients, with a similar distribution in both genders. One of the most significant differences by gender was detected in the GHQ-28 score, where 32.2% of the men scored ≥ 6 compared to 42.9% of the women (*P *< .001) (Table 1).

**Table 1:** Characteristics of the patients for each gender at baseline.

*SOCIODEMOGRAPHIC VARIABLES*	MEN	WOMEN	P
*Age (Mean SD)*	N = 112	N = 63	0.11
	67.11(10.9)	69.76(9.9)	

*Age group*	N = 110	N = 62	0.55
< = 60	29(25.9)	12(19)	
61-70	38(33.9)	22(34.9)	
> 70	43(38.4)	28(44.4)	

	N = 112 n (%)	N = 63 n (%)	

*Educational Levels*			0.01
Illiterate - No Educational Level completed	45(40.2)	37(58.7)	
Primary	41(36.6)	22(34.9)	
Secondary and University	26(23.2)	4(6.3)	

*CLINICAL VARIABLES*			

*Diagnostic group*			0.84
Acute myocardial infarction	48(42.9)	26(41.3)	
Unstable angina	64(57.1)	37(58.7)	

*GHQ-28 (baseline)*			0.01
< 6	86(76.8)	36(57.1)	
≥ 6	26(23.2)	27(42.9)	
GHQ-28 (Mean SD)	3.04(4.1)	6.95(6.02)	0.00

*Tobacco*			0.00
Smoker	41(36.6)	2(3.2)	
Non-smoker	71(63.4)	61(96.8)	

*Diabetes*			
Yes	39(34.8)	30(47.6)	0.10

*Hypercholesterolemia*			
Yes	47(42)	26(41.3)	0.93

*High blood pressure*			
Yes	59(52.7)	43(68.3)	0.05

*Obesity (BMI≥30)*			
Yes	26(23.2)	26(41.3)	0.01

*Physical Activity*			
Yes	60(53.6)	20(31.7)	0.01

*Previous history of CHD*.			
Yes	65(58)	34(54.0)	0.60

*Comorbidity*			
Yes	70(62.5)	40(63.5)	0.90

*Ejection fraction*			
< 50	32(28.6)	12(19)	0.16
≥50	80(71.4)	51(81)	

*Risk factor*			0.80
None	12(10.7)	6(9.5)	
One or more	100(89.3)	57(90.5)	

BMI: Body mass index CHD: Coronary Heart Disease

**Figure 1:**
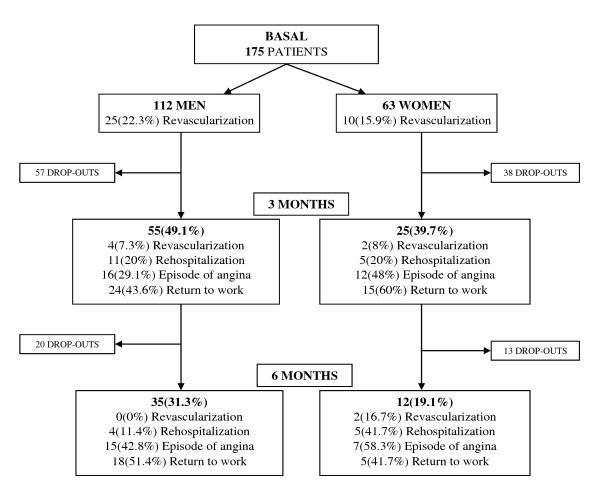
**Flowchart of participants throughout the study**.

Figure 1 shows that, of the patients who began the study, 55 men (49.1%) and 25 women (39.7%) remained at 3 months, and 35 men (31.3%) and only 12 women (19.1%) at 6 months. However, there were no significant differences in socio-demographic and clinical characteristics between the responding and non-responding patients at 3 and at 6 months, except that the women who remained in the study were younger (73 vs 65 years at 3 months, *P *< .05; 71 vs 65 years at 6 months, *P *< .05).

Figure 1 also shows that the percentage of men who had undergone revascularization at baseline was higher than that of women, but a higher percentage of women underwent this operation at a later time (3 to 6 months (*P *= 0.01)). It is also worth pointing out that the women had more episodes of angina throughout the whole of follow-up, but this difference was not statistically significant; they were also rehospitalised more often between 3 and 6 months (*P *= 0.02).

Regarding the differences in the baseline scores in the dimensions of the SF-36, the men remaining in the study at 3 months had lower scores only in the PF dimension when compared with the patients who dropped out during follow-up. On the other hand, the women who remained at 6 months had lower baseline scores in the VT, SF and MCS dimensions.

The raw analysis of the differences between each of the eight specific and the two summary dimensions of the SF-36 during follow-up shows a significant improvement among the men in the scores obtained in the BP and GH dimensions. However, the rest of the dimensions showed no significant changes throughout follow-up. (Table 2)

**Table 2:** Mean (SD) of SF-36 dimensions in Spanish population and study population during follow-up by gender

	MEN							WOMEN						
	
	BASELINE N = 112		3 MONTHS N = 55		6 MONTHS N = 35		*p*	BASELINE N = 63		3 MONTHS N = 25		6 MONTHS N = 12		*p*
				
	Mean	SD	Mean	SD	Mean	SD		Mean	SD	Mean	SD	Mean	SD	
PF	69.4	25.1	74.3	25.7	72.4	25.1	0.45	40.0	29.3	45.4	23.2	37.1	28.5	0.19

RP	62.8	47.9	73.6	41.5	74.3	41.8	0.31	25.0	39.9	62.5	43.3	68.7	46.6	0.02**

BP	64.5	27.6	67.3	25.5	76.5	22.1	0.02**	33.8	20.7	40.9	23.7	41.7	15.9	0.45

GH	62.1	17.5	69.2	17.1	69.6	16.0	0.02*	45.8	22.4	55.6	23.1	53.5	20.9	0.43

VT	69.7	27.1	73.4	26.5	74.7	21.8	0.31	30.8	21.1	58.7	28.3	49.6	31.7	0.003**

SF	86.9	26.2	85.0	29.7	87.5	29.1	0.89	38.5	30.8	80.2	24.7	71.8	31.6	0.006**

RE	88.6	26.7	91.4	28.4	90.5	28.7	0.79	41.7	51.5	47.2	48.1	66.7	49.2	0.42

MH	70.2	17.1	73.3	17.5	72.5	13.8	0.34	49.7	13.4	52.0	25.0	54.0	21.8	0.93

PCS	43.2	11.6	45.8	10.2	46.9	10.6	0.06	33.3	10.8	40.6	7.4	37.5	11.1	0.08

MCS	52.3	7.7	52.5	9.9	52.2	7.5	0.97	35.3	9.4	41.9	14.3	44.4	13.1	0.78

PF: physical functioning; RP: role physical; BP: body pain; GH: general health. PCS: physical component summary; VT: vitality; SF: social functioning; RE: role emotional; MH: mental health; MCS: mental component summary.

ANOVA test with the Bonferroni test for *post hoc *comparisons in men and Friedman test with the Wilkoxon test for *post hoc *comparison in women.

p: comparison at three times.

* statistically significant difference between baseline and 3 months

** statistically significant difference between baseline and 6 months.

No significant differences were observed between 3 months and 6 months in any dimension

The analysis of the raw data for women showed a significant improvement during follow-up in the scores obtained in the RP, and VT dimensions, while this was particularly so in the SF dimension (baseline score of 38.5 vs 80.2 at 3 months). However, no differences were observed in the remaining dimensions of the SF-36. (Table 2).

It is also necessary to point out that the baseline scores were lower for women than for men in all the dimensions of the SF-36. These differences were all statistically significant.

### Analysis of the evolution of HRQL with GEE models

#### Men

In the analysis of the evolution of HRQL in men, the MCS dimension was not considered worth studying because the scores remained constant during follow-up (Table 2) and they were very close to those of the Spanish reference population (mean 50, SD:10).

The analysis of the PF, BP, SF, and PCS dimensions revealed that, while scores remained unchanged at 3 months, at 6 months patients had made a significant recovery. On the contrary, the RE dimension score only improved at 3 months; a significant increase was observed in the GH dimension at both 3 and 6 months (Table 3).

Despite not showing changes during follow-up, the RP, VT and MH dimensions were also analysed to identify the factors which conditioned the lack of improvement over time.

Among the factors associated with HRQL, the patients' age was shown to have a negative effect on the evolution of the PF, RP, BP and PCS dimensions. Also, the patients who were rehospitalised or suffered an episode of angina between 3 and 6 months of the follow-up showed worse evolution in some dimensions of their quality of life. Furthermore, a history of coronary disease had a negative effect on the evolution of the GH and PCS dimensions, while the presence of comorbidity and cardiovascular risk factors negatively affected RP and BP, respectively (Table 3).

**Table 3:** Generalized Estimating Equation models for MEN of SF-36.

*PHYSICAL DIMENSIONS*					
	***VARIABLES***	**B**	**SE**	**C.I. (95%)**	**Sig.**

	*Age*	-0.61	0.18	(-0.97; -0.26)	.001
	
*PHYSICAL FUNCTIONING* *(PF)*	*Frequency of angina^1 ^*(≥ 1 between baseline-3 months) *Frequency of angina *(≥ 1 between 3 and 6 months)	-8.02 -11.02	6.99 5.68	(-21.73; 5.69) (-22.16; 0.12)	.251 .052
	
	*GHQ-28*	-3.47	0.53	(-4.51; -2.44)	000
	
	Time 1^5^ Time 2	6.64 8.82	3.57 3.72	(-0.35; 13.63) (1.53; 16.12)	.062 .018

	*Age*	-0.82	0.24	(-1.29; -0.36)	.001
	
	*Comorbidity (Yes)*	-14.64	5.11	(-24.66; -4.62)	.004
	
*ROLE PHYSICAL* *(RP)*	*Rehospitalization^2 ^*(Between baseline-3 months) *Rehospitalization *(Between 3 and 6 months)	20.60 -45.09	7.07 15.37	(6.75; 34.45) (-75.20; -14.97)	.004 .003
	
	*GHQ-28*	-5.51	0.76	(-6.99; -4.03)	.000
	
	Time 1^5^ Time 2	-4.83 2.07	6.04 6.76	(-11.17; 15.31) (-16.67; 7.02)	.424 .759

	*Age*	-0.71	0.14	(-0.98; -0.44)	.000
	
	*Risk Factor *(Yes)	-13.15	5.07	(-23.09; -3.21)	.010
	
*BODY PAIN* *(BP)*	*Frequency of angina^1 ^*(≥ 1 between baseline-3 months) *Frequency of angina *(≥ 1 between 3 and 6 months)	-3.25 -14.01	6.55 5.24	(-16.08; 9.58) (-24.29; -3.74)	.620 .007
	
	*GHQ-28*	-2.77	0.63	(-4.01; -1.54)	.000
	
	Time 1^5^ Time 2	4.43 14.16	3.69 4.56	(-2.80; 11.67) (5.23; 23.10)	.230 .002

	*Previous history of CHD *(Yes)	-5.50	2.49	(-10.37; -0.63)	.027
	
*GENERAL HEALTH* *(GH)*	*GHQ-28*	-2.13	0.32	(-2.76; -1.50)	.000
	
	Time 1^5^ Time 2	4.64 5.16	2.21 2.36	(0.30; 8.97) (0.54; 9.78)	.036 .029

	*Age*	-0.25	0.05	(-0.35; -0.14)	.000
	
	*Previous history of CHD *(Yes)	-3.29	1.33	(-5.88; -0.69)	.013
	
*PHYSICAL COMPONENT SUMMARY* *(PCS)*	*Rehospitalization^2 ^*(Between baseline-3 months) *Rehospitalization *(Between 3 and 6 months)	3.68 -9.79	1.94 3.93	(-0.12; 7.48) (-17.48; -2.10)	.058 .013
	
	*GHQ-28*	-1.46	0.22	(-1.90; -1.02)	.000
	
	Time 1^5^ Time 2	0.67 3.44	1.32 1.43	(-1.92; 3.27) (0.64; 6.24)	.610 .016

*MENTAL DIMENSIONS*					

	**VARIABLES**	**B**	**SE**	**C.I. (95%)**	**Sig.**

	*Comorbidity (Yes)*	-9.53	2.82	(-15.06; -3.99)	.001
	
*VITALITY* *(VT)*	*GHQ-28*	-3.75	0.52	(-4.77; -2.74)	.000
	
	Time 1^5^ Time 2	-0.88 -0.87	3.17 3.38	(-7.09; 5.33) (-7.49; 5.76)	.781 .797

	*Age*	-0.54	0.18	(-0.89; -0.19)	.002
	
SOCIAL FUNCTIONING (SF)	*Frequency of angina^1 ^*(≥ 1 between baseline-3 months) *Frequency of angina *(≥ 1 between 3 and 6 months)	-22.94 -21.62	9.74 7.86	(-42.04; -3.85) (-37.02; -6.22)	.019 .006
	
	*GHQ-28*	-2.87	0.57	(-3.98; -1.75)	.000
	
	Time 1^5^ Time 2	3.09 8.10	3.74 2.75	(-4.24; 10.42) (2.71; 13.49)	.409 .003

*ROLE EMOTIONAL* *(RE)*	*Frequency of angina^1 ^*(≥ 1 between baseline-3 months) *Frequency of angina *(≥ 1 between 3 and 6 months)	-15.42 6.46	8.71 9.35	(-32.49; 1.65) (-11.87; 24.80)	.077 .489
	
	*GHQ-28*	-2.20	0.80	(-3.76; -0.64)	.006
	
	Time 1^5^ Time 2	10.90 -0.68	3.09 7.26	(4.84; 16.96) (-14.90; 13.55)	.000 .926

	*Revascularization^3 ^*(Baseline) *Revascularization *(Between baseline-3 months)	-1.97 20.91	4.51 8.26	(-10.81; 6.87) (4.72; 37.10)	.662 .011
	
*MENTAL HEALTH* *(MH)*	*Frequency of angina^1 ^*(≥ 1 between baseline-3 months) *Frequency of angina *(≥ 1 between 3 and 6 months)	-12.68 -4.64	4.95 3.55	(-22.39; -2.97) (-11.59; 2.31)	.010 .191
	
	*Return to work^4 ^*(Between baseline-3 months) *Return to work *(Between 3 and 6 months)	7.48 1.46	3.69 3.95	(0.25; 14.71) (-6.28; 9.21)	.043 .711
	
	*GHQ-28*	-1.83	0.51	(-2.84; -0.83)	.000
	
	Time 1^5^ Time 2	-3.39 -1.61	3.10 3.57	(-9.46; 2.68) (-8.61; 5.38)	.274 .652

^1 ^Reference Category: No episode of angina occurred during the whole period

^2^Reference Category: No rehospitalization occurred during the whole period

^3^Reference Category: No revascularization occurred during the whole period

^4^Reference Category: No return to work occurred during the whole period

^5^Reference Category: Time 0: Baseline

Time 1: 3 months

Time 2: 6 months

As for the mental dimensions, the frequency of angina stands out as the variable having the most negative effect on SF, RE and MH (Table 3).

Lastly, it should be emphasized that the patients' mental health, measured with the GHQ-28, was the only variable associated with deterioration in all the dimensions of HRQL throughout the study. It caused a decrease of between 1.4 and 5.6 points in HRQL for every unit increase in the GHQ-28 during follow-up (Table 3).

#### Women

In the analysis of the evolution of HRQL in women, the scores for the RP, SF, and PCS dimensions remained unchanged at 3 months. However, they were higher at 6 months of follow-up (Tables 4 and 5).

**Table 4:** Generalized Estimating Equation models for WOMEN for Physical Dimensions of SF-36.

	*VARIABLES*	B	SE	C.I. (95%)	Sig.
	*Previous history of CHD *(Yes)	-18.33	4.34	(-26.84; -9.81)	.000
	
	*Diagnostic group *(AMI)	14.52	4.46	(5.78; 23.27)	.001
	
	*Comorbidity *(Yes)	-9.64	4.51	(-18.48; -0.80)	.033
	
*PHYSICAL FUNCTIONING* *(PF)*	*Rehospitalization^1 ^*(Between baseline3 months) *Rehospitalization *(Between 3 and 6 months)	-9.41 -18.07	6.62 7.47	(-22.38; 3.56) (-32.72; -3.43)	.155 .016
	
	*GHQ-28*	-1.70	0.35	(-2.38; -1.01)	.000
	
	Time 1^4^ Time 2	0.01 1.23	4.16 6.21	(-8.14; 8.16) (-10.95; 13.40)	.998 .844

	*Age*	-1.25	0.49	(-2.22; -0.28)	.011
	
*ROLE PHYSICAL* *(RP)*	*Frequency of angina^2 ^*(≥1 between baseline-3 months) *Frequency of angina *(≥1 between 3 and 6 months)	-12.40 -27.15	14.72 16.67	(-41.25; 16.45) (-59.82; 5.51)	.400 .103
	
	*GHQ-28*	-4.36	0.55	(-5.44; -3.29)	000
	
	Time 1^4^ Time 2	4.81 30.81	12.83 10.23	(-20.33; 29.95) (10.76; 50.86)	.708 .003

*BODY PAIN* *(BP)*	*Previous history of CHD *(Yes)	-25.50	5.27	(-35.82; -15.18)	.000
	
	*GHQ-28*	-0.99	0.46	(-1.90; -0.09)	.031
	
	Time 1^4^ Time 2	-0.50 1.98	5.44 5.21	(-11.15; 10.15) (-8.24; 12.19)	.927 .705

*GENERAL HEALTH* *(GH)*	*Revascularization^3 ^*(Baseline) *Revascularization *(Between baseline and 3 months) *Revascularization *(Between 3 and 6 months)	-10.12 7.16 -17.07	6.48 9.84 8.32	(-22.82; 2.59) (-12.12; 26.44) (-33.38; -0.77)	.119 .466 .040
	
	*GHQ-28*	-2.14	0.33	(-2.78; -1.49)	.000
	
	Time 1^4^ Time 2	0.20 -1.97	4.06 4.02	(-7.75; 8.15) (-9.85; 5.91)	.961 .624

	*Previous history of CHD *(Yes)	-8.32	1.98	(-12.20; -4.45)	.000
	
	*Diagnostic group *(AMI)	4.52	2.12	(0.36; 8.68)	.033
	
*PHYSICAL COMPONENT SUMMARY* *(PCS)*	*Frequency of angina^2 ^*(≥1 between baseline-3 months) *Frequency of angina *(≥1 between 3 and 6 months)	0.83 -10.58	3.06 2.72	(-5.17; 6.83) (-15.91; -5.24)	.786 .000
	
	*GHQ-28*	-0.57	0.15	(-0.86; -0.27)	.000
	
	Time 1^4^ Time 2	2.46 8.80	2.47 1.68	(-2.38; 7.30) (5.51; 12.09)	.319 .000

^1^Reference Category: No rehospitalisation occurred during the whole period

^2^Reference Category: No episode of angina occurred during the whole period

^3^Reference Category: No revascularization occurred during the whole period

^4^Reference Category: Time 0: Baseline

Time 1: 3 months

Time 2: 6 months

**Table 5:** Generalized Estimating Equations models for WOMEN for Mental Dimensions of SF-36.

	*VARIABLES*	B	SE	C.I. (95%)	Sig.
	*Educational Level *(Primary) *Educational Level *(Secondary and University)	0.63 13.23	4.57 5.00	(-8.32; 9.58) (3.44; 23.03)	.890 .000
	
	*Previous history of CHD *(Yes)	-11.39	4.63	(-20.46; -2.32)	. 014
	
*VITALITY* *(VT)*	*Revascularization^1 ^*(Baseline) *Revascularization *(Between baseline-3 months) *Revascularization *(Between 3 and 6 months)	4.38 26.16 -39.69	11.06 19.88 9.41	(-17.30; 26.07) (-12.82; 65.12) (-58.13; -21.25)	.692 .188 .000
	
	*GHQ-28*	-2.98	0.31	(-3.58; -2.37)	.000
	
	Time 1^4^ Time 2	0.56 5.60	5.19 5.88	(-9.62; 10.73) (-5.93; 17.12)	.915 .341

	*Educational Level *(Primary) *Educational Level *(Secondary and University)	1.09 30.26	6.57 7.47	(-11.78; 13.96) (15.62; 44.91)	.869 .000
	
	*Previous history of CHD *(Yes)	-23.40	7.05	(-37.22; -9.58)	. 001
	
*SOCIAL FUNCTIONING* *(SF)*	*Revascularization^1 ^*(Baseline) *Revascularization *(Between Baseline-3 months) *Revascularization *(Between 3 and 6 months)	10.40 5.67 -28.37	11.82 29.80 26.63	(-12.76; 33.56) (-52.73; 64.08) (-80.57; 23.83)	.379 .849 .287
	
	*GHQ-28*	-2.26	0.52	(-3.28; -1.24)	.000
	
	Time 1^4^ Time 2	8.40 18.00	6.27 8.88	(-3.88; 20.69) (0.60; 35.41)	.180 .043

*ROLE EMOTIONAL (RE)*	*Revascularization^1 ^*(Baseline) *Revascularization *(Between baseline-3 months) *Revascularization *(Between 3 and 6 months)	-0.65 -37.27 -11.53	16.70 8.33 27.03	(-33.37; 32.08) (-53.59; -20.95) (-64.50; 41.44)	.969 .000 .670
	
	*GHQ-28*	-3.53	0.60	(-4.70; -2.36)	.000
	
	Time 1^4^ Time 2	-12.39 1.28	8.72 15.61	(-29.48; 4.69) (-29.32; 31.87)	.155 .935

	*Previous history of CHD *(Yes)	-8.21	3.16	(-14.40; -2.02)	.009
	
*MENTAL HEALTH* *(MH)*	*Rehospitalization^2 ^*(Between baseline-3 months) *Rehospitalization *(Between 3 and 6 months)	-6.75 -21.69	3.47 9.95	(-13.55; 0.05) (-41.19; -2.18)	.052 .029
	
	*GHQ-28*	-1.88	0.28	(-2.43; -1.34)	.000
	
	Time 1^4^ Time 2	-1.41 6.42	3.30 4.63	(-7.87; 5.05) (-2.65; 15.49)	.669 .165

*MENTAL COMPONENT SUMMARY* *(MCS)*	*Revascularization^1 ^*(Baseline) *Revascularization *(Between baseline-3 months) *Revascularization *(Between 3 and 6 months)	2.70 -3.07 -25.75	3.28 4.58 4.16	(-3.72; 9.13) (-12.05; 5.91) (-33.90; -17.59)	.410 .502 .000
	
	*Frequency of angina^3 ^*(≥1 between baseline-3 months) *Frequency of angina *(≥1 between 3 and 6 months)	-4.40 18.21	3.67 5.27	(-11.60; 2.79) (7.87; 28.54)	.230 .001
	
	*GHQ-28*	-1.34	0.152	(-1.64; -1.04)	.000
	
	Time 1^4^ Time 2	-0.04 -6.97	2.41 4.50	(-4.77; 4.69) (-15.79; 1.84)	.986 .121

^1^Reference Category: No revascularization occurred during the whole period

^2^Reference Category: No rehospitalisation occurred during the whole period

^3^Reference Category: No episode of angina occurred during the whole period

^4^Reference Category: Time 0: Baseline

Time 1: 3 months

Time 2: 6 months

Despite not showing changes, the other dimensions were analyzed to identify the factors which conditioned the lack of improvement over time.

The analysis of the factors affecting the evolution of HRQL in the female population highlighted that, like men, a worse mental health, measured with the GHQ-28, affected all the dimensions of the SF-36; also, a history of coronary disease led to a decrease in the scores in most dimensions of the SF-36 (Tables 4 and 5).

Being rehospitalised, suffering, an episode of angina, or undergoing revascularization were also associated with lower scores in the PF, GH, and PCS dimensions in the second period of follow-up. Furthermore, the women diagnosed with AMI showed better evolution in the PF and PCS dimensions than those with unstable angina (Table 4).

Lastly, the women who had undergone revascularization during follow-up showed worse evolution in the VT, RE, and MCS dimensions than those who had not; and among the women who had been rehospitalised, MH became worse over the duration of the study (Table 5).

## Discussion

The role of gender differences in HRQL in coronary patients is controversial, so understanding the specific determinants of HRQL in men and women might be of clinical importance in, for example, follow-up or rehabilitation programs after a heart attack. This study was carried out to know the gender differences in the evolution of HRQL at 3 and 6 months after a coronary event, and to identify the key clinical, demographic, and psychological characteristics associated with these changes in each gender. The following results stand out: a) the greater number of men who underwent revascularization during admission and the fact that the women were operated on at a later time (between 3 and 6 months); b) the greater frequency of rehospitalization among the women between 3 and 6 months; c) the baseline scores in the SF-36 were lower among women. d) The men showed better evolution at 6 months in most of the physical dimensions, and Social Functioning. However, the women only improved in Physical Component Summary, Role Physical, and Social Functioning at 6 months. e) the variables most associated with unfavourable evolution of HRQL in the men were deterioration in mental health and angina frequency. Mental health was also a determining factor in the evolution of the women's quality of life, although this was also affected by other variables, such as a clinical history of the disease, undergoing revascularization during the second period, and angina frequency.

Most studies into HRQL in coronary patients suggest that women do not cope as well physically and psychosocially as men. However, the literature is not consistent, and it remains unclear why gender-related differences in HRQL exist among coronary patients [29]. The effect of older age or a greater frequency of comorbidity among women, or a greater tendency to carry out surgical procedures such as revascularization on men have been highlighted. In our study, comorbidity was not really a determining variable for the patients' HRQL as it only affected two dimensions of the SF-36 in the men. Likewise, the presence of risk factors did not have a special impact on either gender. On the other hand, revascularization was identified as a factor affecting the quality of life of the female population, especially when this was carried out during the second period of the study. In this respect, it is worth highlighting that revascularization was carried out earlier in the men, which may have conditioned the worse evolution observed in the women, who suffered higher frequencies of angina and rehospitalization during follow-up. Hemingway et al [30] and Aguado-Romero and co-workers [31] detail the tendency to operate less on women with coronary disease than on men, although the latter try to justify these differences by referring to limitations in their data. Likewise, Willingham and Kilpatrick [32] find evidence that women seem less likely to be diagnosed with an acute myocardial infarction (AMI) in the first place, despite a raised cTNT (cardiac Troponin T) value being a completely objective finding available to the clinician. The author explains that the reasons for this finding appear to be independent of the older age at which the females were affected in their study. Thus, other factors, such as the perception that women have a lower pre-test probability of infarction, may influence the clinician's discharge decision [32]. The different attitude to treatment among women may be another determining factor.

On the other hand, several studies have found that the evolution of HRQL differs between men and women after coronary surgery. Bute et al [33] concluded that women do not obtain the same benefit from CABG (Coronary Artery Bypass Grafting) surgery as men, and that the difference cannot be attributed to preoperative divergence. One possible explanation for this is that women's compromised HRQL is less related to cardiac health than men's, with other environmental and/or personality variables related to quality of life affecting women more than men [33].

One relevant aspect of the study which confirms the hypothesis formulated is the effect of the deterioration of mental health on the evolution of all the dimensions of HRQL, both in men and women. Previous studies have demonstrated substantial rates of depression during the first year after a myocardial infarction [10] and several authors found that anxiety and depression predicted HRQL 12 months after AMI [1,34]. Brink et al reported depression after an AMI as a common determinant of a lower PCS score in both men and women, but their study did not include any measures of comorbidity or clinical parameters [1]. In our study, mental health affected the evolution of the HRQL of both the men and women when adjusted for other variables. However, a higher percentage of the women than the men had baseline scores below 6 in the GHQ-28. This may indicate that women deal worse with the disease from the outset, but it is not possible to confirm this as mental health was not assessed prior to the coronary event.

In our study, the women's HRQL was lower than the men's at baseline. Likewise, while the men's HRQL improved at 6 months in nearly all the physical dimensions, and in Social Functioning, the women only improved in Role Physical, Physical Component Summary, and Social Functioning. This is partly in accordance with the results obtained by Emery and co-workers, [17] who show that men and women have increased scores in physical health over time, but women have significantly lower scores in physical dimensions across all assessments.

Lastly, a limitation of our study is the small sample size, due to drop-outs during follow-up. However, the small differences observed between the drop-outs and the patients that remained indicate that selection bias was minimal. Likewise it is also necessary to explain that although the patients were contacted three times before being considered drop-outs, their being given an appointment in the hospital's Department of Preventive Medicine, and not in the Department of Cardiology, could have influenced the number of drop-outs observed.

Another limitation in this study was that no information was collected regarding the outcome status of patients lost during follow-up. However, some informal data were explored revealing no particular selection bias.

On the other hand, a strength of the study is that it uses a method of analysis which is rarely used in healthcare research. This makes it possible to know the independent effect of the different variables on the evolution of HRQL, with a more accurate estimation of the parameters than traditional regression methods [27,35,36].

## Conclusions

There are differences between the evolution of the HRQL of the men and women in the study after a coronary attack. Mental health was the determinant most frequently associated with HRQL in both genders. However, other clinical determinants of HRQL differed between genders, emphasizing the importance of individualizing the intervention and the content of rehabilitation programs. Likewise, the recognition and treatment of mental disorders in these patients could be crucial.

## Competing interests

The authors declare that they have no competing interests.

## Authors' contributions

MD has made substantial contributions to the conception and design of the paper and the analysis and interpretation of data. She has been involved in drafting the manuscript.

CR has participated in patient evaluations, data collection, and the analysis of data. RA has made substantial contributions to the clinical evaluation of the patients and the coordination of the hospital work. IF has made substantial contributions to the conception and design of the paper. She has been involved in revising the manuscript and she has given her final approval of the version to be sent. All the authors have read and approved the final manuscript.

## Pre-publication history

The pre-publication history for this paper can be accessed here:

http://www.biomedcentral.com/1471-2261/11/24/prepub
